# Engineering the Erythromycin-Producing Strain *Saccharopolyspora erythraea* HOE107 for the Heterologous Production of Polyketide Antibiotics

**DOI:** 10.3389/fmicb.2020.593217

**Published:** 2020-12-08

**Authors:** Jin Lü, Qingshan Long, Zhilong Zhao, Lu Chen, Weijun He, Jiali Hong, Kai Liu, Yemin Wang, Xiuhua Pang, Zixin Deng, Meifeng Tao

**Affiliations:** ^1^State Key Laboratory of Microbial Metabolism, Joint International Research Laboratory of Metabolic and Developmental Sciences, Shanghai-Islamabad-Belgrade Joint Innovation Center on Antibacterial Resistances, School of Sciences and Biotechnology, Shanghai Jiao Tong University, Shanghai, China; ^2^State Key Laboratory of Microbial Technology, School of Life Sciences, Shandong University, Jinan, China

**Keywords:** *Saccharopolyspora erythraea*, CRISPR/Cas9-CodA(sm), heterologous expression, polyketides, antibiotic production

## Abstract

Bacteria of the genus *Saccharopolyspora* produce important polyketide antibiotics, including erythromycin A (*Sac. erythraea*) and spinosad (*Sac. spinosa*). We herein report the development of an industrial erythromycin-producing strain, *Sac*. *erythraea* HOE107, into a host for the heterologous expression of polyketide biosynthetic gene clusters (BGCs) from other *Saccharopolyspora* species and related actinomycetes. To facilitate the integration of natural product BGCs and auxiliary genes beneficial for the production of natural products, the erythromycin polyketide synthase (*ery*) genes were replaced with two bacterial *attB* genomic integration sites associated with bacteriophages ϕC31 and ϕBT1. We also established a highly efficient conjugation protocol for the introduction of large bacterial artificial chromosome (BAC) clones into *Sac. erythraea* strains. Based on this optimized protocol, an arrayed BAC library was effectively transferred into *Sac. erythraea*. The large spinosad gene cluster from *Sac. spinosa* and the actinorhodin gene cluster from *Streptomyces coelicolor* were successfully expressed in the *ery* deletion mutant. Deletion of the endogenous giant polyketide synthase genes *pkeA1*-*pkeA4*, the product of which is not known, and the flaviolin gene cluster (*rpp*) from the bacterium increased the heterologous production of spinosad and actinorhodin. Furthermore, integration of pJTU6728 carrying additional beneficial genes dramatically improved the yield of actinorhodin in the engineered *Sac. erythraea* strains. Our study demonstrated that the engineered *Sac. erythraea* strains SLQ185, LJ161, and LJ162 are good hosts for the expression of heterologous antibiotics and should aid in expression-based genome-mining approaches for the discovery of new and cryptic antibiotics from *Streptomyces* and rare actinomycetes.

## Introduction

Bioactive natural products isolated through actinomycete fermentation processes are important sources of therapeutics and agrochemicals, including antibacterials (e.g., erythromycin A, tylosin, and vancomycin); antifungals (e.g., amphotericin B); immunosuppressants (e.g., FK-506 and rapamycin); anticancer agents (e.g., doxorubicin and epoxomicin); anthelmintics (e.g., avermectin); and insecticides (e.g., spinosad) (Challis, 2014; Pham et al., 2019). Most of these compounds are isolated from the most dominant actinomycete genus, *Streptomyces*. However, several important compounds come from non-*Streptomyces* actinomycetes, known as rare actinomycetes, such as vancomycin (*Amycolatopsis orientalis*), erythromycin (*Saccharopolyspora erythraea*), and spinosad (*Saccharopolyspora spinosa*) (Pham et al., 2019). Indeed, rare actinomycetes have been regarded as a storehouse of novel antibiotics (Tiwari and Gupta, 2012), and novel natural products are increasingly discovered from among this group (Nett et al., 2009; Choi et al., 2015).

Genome sequencing has revealed that each actinomycete genome usually harbors more than ten biosynthetic gene clusters (BGCs) for different secondary metabolites, and a majority of these BGCs are silent or cryptic under normal laboratory cultivation conditions (Nett et al., 2009; Choi et al., 2015). Heterologous biosynthesis has emerged as a viable route to access the beneficial properties of cryptic natural products. Intact BGC of interest is cloned into a suitable vector and delivered into a heterologous host for optimal production of the unknown compound, using the host’s gene expression machinery, precursor substances, and cofactors. Bioinformatics tools, such as antiSMASH, NP.searcher, and ClustScan, are available for the prediction of BGCs having the potential to synthesize novel compounds (Starcevic et al., 2008; Li et al., 2009; Blin et al., 2019). Various techniques, for example, the transformation-associated recombination system (Kouprina and Larionov, 2016), integrase-mediated recombination system (Olorunniji et al., 2019), and bacterial artificial chromosome (BAC) system (Sosio et al., 2000), have been adapted for cloning some large, intact BGCs. However, the current heterologous hosts of actinomycetes mostly belong to the *Streptomyces* genus, including *Streptomyces coelicolor* (Gomez-Escribano and Bibb, 2011)*; Streptomyces lividans* (Xu et al., 2016, 2020; Zhao et al., 2016; Gao et al., 2017; Chen et al., 2018; Peng et al., 2018)*; Streptomyces avermitilis* (Komatsu et al., 2013)*;* and *Streptomyces albus* (Chater and Wilde, 1976; Myronovskyi et al., 2018). Few heterologous hosts are derived from rare actinomycetes, mainly due to the lack of efficient genetic manipulation systems that would enable the substantial strain engineering required for removing internal competitive biosynthetic pathways, such as polyketide BGCs (Pfeifer and Khosla, 2001).

Site-specific recombination systems enable one to construct recombinant plasmids in an experimentally tractable host such as *Escherichia coli* and then transfer the plasmids into recipients by conjugation (Flett et al., 2006). The well-characterized ϕC31 and ϕBT1 attachment/integration (*att*/*int*) systems have been engineered into many integrative *Streptomyces* plasmids, allowing the efficient integration of large plasmids into the highly conserved and relatively neutral *attB* sites in *Streptomyces* genomes (Zhang et al., 2016). Many actinomycetes, such as *Sac. erythraea*, lack a typical *attB* site, which has largely impeded efforts for the delivery of exogenous DNA. Indeed, previous studies have introduced an *attB^ϕ*C*31^* site into the genome of *Sac. erythraea* for the purpose of improving production of erythromycin by transferring exogenous or endogenous genes (Rodriguez et al., 2003; Wu et al., 2011). In another approach to strain engineering, the entire spinosad BGC was assembled using a multi-step homologous recombination procedure and was used to replace the native erythromycin BGC in the wild-type strain *Sac. erythraea* ATCC 40137 (Huang et al., 2016); through substantial genetic engineering and mutagenesis, this heterologous expression system allowed spinosad production to reached a titer of 800 mg/L in the resulting *Sac. erythraea* strain, which is hundreds of times higher than those in *Streptomyces* hosts, including *S. albus*, *S. coelicolor*, and *S. lividans* (Huang et al., 2016; Tan et al., 2017; Zhao et al., 2017). These studies confirmed the suitability of *Saccharopolyspora* spp. for expression of heterologous BGCs from related actinobacterial species, and also suggested the necessity of non-*Streptomyces* actinomycete hosts for heterologous expression-based genome mining.

In this study, we engineered an erythromycin-producing strain of *Saccharopolyspora* into a suitable heterologous host for the expression of large size polyketide BGCs, by optimizing the genetic manipulation system, deleting background polyketide biosynthetic pathways, and other modifications of the host genome.

## Materials and Methods

### Strains, Plasmids, and Culture Conditions

Strains used in this study are listed in Table 1. *Sac. erythraea* HOE107 and its derivatives were grown on ESM medium at 34°C for spore preparation as previously described (Li et al., 2013). *E. coli* strains, *Staphylococcus aureus*, and *Bacillus mycoides* were cultured in Luria-Bertani (LB) medium at 37°C. The following antibiotics were added to the medium when required: apramycin (50 μg/mL), hygromycin (100 μg/mL), spectinomycin (50 μg/mL), ampicillin (100 μg/mL), kanamycin (50 μg/mL), chloramphenicol (25 μg/mL). *Sta. aureus* and *B. mycoides* were used as indicator strains in the bioassay experiments. *E. coli* XL1-Blue (Stratagene) was used as the host for cosmid library construction, and *E. coli* DH10B (Invitrogen) was used for general cloning, plasmid maintenance, and as host for the BAC library. *E. coli* ET12567/pUB307 was used as a helper strain to mobilize *oriT_*RK*__2_*-plasmids from *E. coli* into *Sac. Erythraea* by tri-parental conjugation (Flett et al., 2006).

**TABLE 1:** Bacterial strains used in this study.

**Strain**	**Description**	**Reference or source**
***Saccharopolyspora erythraea* strains**		
HOE107	Industrial strain	Laboratory stock
SLQ185	Derivative of HOE107 with the *eryAII*-*eryBI* region of the *ery* cluster replaced by the a*ttB*^ϕ^*^*C*31^*-*aadA*-*attB*^ϕ^*^*BT*1^* cassette	This work
LJ161	Derivative of SLQ185 with deletion of the *pke* cluster	This work
LJ162	Derivative of SLQ185 with deletion of the *rpp* cluster	This work
***Escherichia coli* strains**		
DH10B	Host for general cloning and BAC library construction	Invitrogen
XL1-blue MR	Host for cosmid library construction	Stratagene
ET12567/pUB307	Strain for intergeneric conjugation	Flett et al., 2006

pIJ4642 (Kieser et al., 1992) was used as template for PCR amplification of the *aadA* resistance marker. SuperCos 1 (Stratagene) was used to construct the genomic cosmid library of *Sac. erythraea* HOE107. pOJ260 (Bierman et al., 1992) was used as a non-replicating suicide vector for gene replacement in *Sac. erythraea*. pWHU2653, bearing an engineered clustered regularly interspaced short palindromic repeat (CRISPR)/Cas9 combined with the counterselection system CodA(sm) (Zeng et al., 2015), was used as gene deletion vector in *Sac. erythraea*. pHL931 (Zhao et al., 2016) was the vector for the genomic BAC library of *Sac. spinosa* NRRL18395. pJTU6728 is a derivative of the integrative vector pMS82 and carries the integration site *attP* and *int* loci of the *Streptomyces* temperate phage ϕBT1 (Gregory et al., 2003) and contains the transcription factor gene *nusG*, global regulator genes *afsRS*_*cla*_, and the two drug efflux pump genes *mdfA*_*co*_ and *lrmA*_*co*_ (Peng et al., 2018). pJTU6728 was used to improve the production of heterologous antibiotics in *Sac. erythraea*.

### DNA Manipulation

Isolation of DNA and all subsequent manipulations were performed according to standard protocols (Kieser et al., 2000). Primers used in this study are listed in Table 2. BAC and plasmid constructs are listed in Table 3.

**TABLE 2:** Primers used in this study.

**Primer**	**Sequence (5′→3′)^a^**	**Introduced sequence^b^**
aadA-F	**GAGGTGGAGTACGCGCCCGGGGAGCCCAAGGGCACGCCCTGGCACCCGCACCG**GATCAATTCCCCTGCTC	*attB*^ϕ*C*31^
aadA-R	**GGGTGTGGAGCTGGATCATCTGGATCACTTTCGTCAAAAACCTGGTCAAGGACG**CTTGAGTTAAGCCGCGC	*attB*^ϕ*BT*1^
eryT-F	**ACCTGCCGCACATGCCGGACCCGGAACTGCGAGGCCGTCTCACCG**GAGGTGGAGTACGCGCCCGGGG	45-Homo
eryT-R	**ACCAGCCGTTTCACGCGCTCACCCCCAGTCATGCAAACAATTTTC**GGGTGTGGAGCTGGATCATCTG	45-Homo
eryV1-F	AACTCGACGCCGAGGGCATCG	
eryV1-R	AGCGGGTCCTTCTCGTTGTAGT	
eryV2-F	TGATGAGCCCGGACACGCTCAT	
eryV2-R	TGCCGCTGGCGACGAGGCTGT	
eryV3-F	GTCTGACGCTCAGTGGAACG	
eryV3-R	CGTGCCAATCGGATCAGCCGTC	
pkeG-F	GAATGACCCAACACGAAATCGTTTT	
pkeG-R	GATTTCGTGTTGGGTCATTCGCTGG	
pkeU-F	**CCATTAAT**ACCTCGCAGCTCTCCATCCT	*Ase*I
pkeU-R	TACCGTTCGTATAATGTATGCTATACGAAGTTATCCGGCGTGGTAGCCCACTTC	
pkeD-F	ATAACTTCGTATAGCATACATTATACGAACGGTATCGCGCAGCAGCCGCCAGTA	
pkeD-R	**CGCAATTG**CGCTGCTGGACATGTACAAG	*Mfe*I
rppG-F	GTATCGGTGCATGATCTTCTGTTTT	
rppG-R	AGAAGATCATGCACCGATACGCTGG	
rppU-F	**CCATTAAT**CATGGCCGCGCCGATGAAGA	*Ase*I
rppU-R	TACCGTTCGTATAATGTATGCTATACGAAGTTATCACTGCCCGCCGCAGTAGTC	
rppD-F	ATAACTTCGTATAGCATACATTATACGAACGGTAGGGTGGCGGTGAAGGTGTCC	
rppD-R	**CGCAATTG**TGGGTGCTGGTGAACCTGAT	*Mfe*I
pkeV4-F	TGGCCCACGCCCACTTCGTC	
pkeV4-R	ACCAGGCCCGCACCGAGTTC	
pkeV5-F	GCCCGTACTCCTGGTAGTTG	
pkeV5-R	CGTGCTGTTCTCCTCCATCG	
rppV6-F	CGACTGGCTCGACGGAATAG	
rppV6-R	GAGCAGCAGGAGGTTCTTGG	
rppV7-F	CCGACGCCTACGCCTATCTG	
rppV7-R	GGTTCACCCGGTCCACCTTG	
gtt-F1	**GCTCTAGA**GGATCCTAATACGACTCACTATAAAGGCCACCGGCAAGGTCGTGCAGG	*Xba*I
gtt-R1	**GAACTAGT**GCACCCGCCGATGGCCGACCGCATT	*Spe*I
epi-F1	**GCTCTAGA**GGATCCTAATACGACTCACTATAGGGATCAACAACAACTTCACCAGCA	*Xba*I
epi-R1	**GAACTAGT**TGGAGGTGGATGTGAAATCCCTCGG	*Spe*I
GK-F1	**GCTCTAGA**GCGGTGTTCCTGGGGCGGTTG-3	*Xba*I
GK-R1	**GACTAGT**CGTTGGTGTGCTCGGACATCC	*Spe*I

*^*a*^Introduced sequences are in boldface; restriction sites are also underlined. ^*b*^This column describes the characteristics of the introduced sequences. 45-Homo, the PCR product flanked by a 45-bp homologous sequence was used for λ-Red recombination.*

**TABLE 3:** BAC and plasmid constructs used in this study.

**Name**	**Description**	**Reference or source**
SuperCos 1	Cloning vector for construction of the cosmid library	Stratagene
pMD18-T	TA cloning vector	Takara
pOJ260	Cloning vector with *aacC4* (apramycin resistance gene) and *oriT_*RK*__2_*	Bierman et al., 1992
pMS82	Cloning vector with *aacC4*, *oriT_*RK*__2_*, and *int-attP^ϕ*BT*1^*	Gregory et al., 2003
pHL931	BAC vector containing *oriT_*RK*__2_*, *int-attP^ϕ*C*31^*, *aacC4*, and *aadA*	
pWHU2653	Vector for CRISPR/Cas9-based gene replacement in actinobacteria; contains the counterselectable gene *codA*(*sm*)	Zeng et al., 2015
pIJ4642	Contains *aadA* (spectinomycin adenylyltransferase gene)	Kieser et al., 1992
pJTU6728	*int-attP^ϕ*BT*1^* plasmid construct, *hyg*, *nusG*, *lmrA*_*co*_, *mdfA*_*co*_, *nusG*, *afsR*_*cla*_-*afsS*_*cla*_	Peng et al., 2018
pMM1	*int-attP^ϕ*C*31^* plasmid construct containing the intact actinorhodin BGC from *Streptomyces coelicolor*	Zhou et al., 2012
3H2	BAC clone containing the intact spinosad BGC from *Sac. spinosa* NRRL18395	This work
6C9	Cosmid clone containing 41.2 kb of the *ery* cluster	This work
pHLQ1	pMD18-T containing the *attB^ϕ*C*31^-aadA-attB^ϕ*BT*1^* cassette	This work
pHLQ2	Derivative of 6C9 generated by replacement of the *eryAII*-*eryBI* region of the *ery* cluster with the a*ttB*^ϕ^*^*C*31^*-*aadA*-*attB*^ϕ^*^*BT*1^* cassette	This work
pHLQ3	pOJ260-derived construct for *ery* gene replacement. The pHLQ2 *Xba*I-*Spe*I restriction fragment, which includes the 5.1-kb UHA, *attB^ϕ*C*31^-aadA- attB^ϕ*BT*1^* cassette, and 5.4-kb DHA, was inserted into the *Spe*I site of pOJ260.	This work
pHLJ61	An sgDNA targeting the *rpp* BGC was cloned into the *Bae*I site of pWHU2653 to give pHLJ61.	This work
pHLJ62	The 2.2-kb UHA_rpp_ and 2.2-kb DHA_rpp_ sequences, flanked by *Mfe*I and *Ase*I restriction sites, were amplified from genomic DNA of *Sac. erythraea* HOE107, joined by overlapping PCR, and cloned into pMD18-T to give pHLJ62.	This work
pHLJ63	The joined UHA_rpp_ and DHA_rpp_ sequences were excised from pHLJ62 by *Mfe*I and *Ase*I digestion and cloned into pHLJ61 at the restriction sites of *Eco*RI/*Ase*I to give pHLJ63.	This work
pHLJ67	sgDNA for deletion of *pke* cloned into *Bae*I site of pWHU2653 to give pHLJ67	This work
pHLJ68	The 2.1-kb UHA_pke_ and 2.2-kb DHA_pke_ sequences for *pke* deletion, flanked by *Mfe*I and *Ase*I restriction sites, were amplified from genomic DNA of *Sac. erythraea* HOE107, joined by PCR, and cloned into pMD18-T to give pHLJ68.	This work
pHLJ69	The 2.1-kb UHA_pke_ and 2.2-kb DHA_pke_ sequences for *pke* deletion were excised by *Mfe*I and *Ase*I, and inserted into the *Eco*RI/*Ase*I sites of pHLJ67 to give pHLJ69.	This work
pHL801	*gtt* sequences, flanked by *Xba*I and *Spe*I restriction sites, were amplified from genomic DNA of *Sac. spinosa* NRRL18395 and cloned into pGEM-T.	Guo et al., 2012
pHL802	*epi* sequences, flanked by *Xba*I and *Spe*I restriction sites, were amplified from genomic DNA of *Sac. spinosa* NRRL18395 and cloned into pGEM-T.	Guo et al., 2012
pHLJ810	*gdh-kre* sequences, flanked by *Xba*I and *Spe*I restriction sites, were amplified from genomic DNA of *Sac. spinosa* NRRL18395 and cloned into pMD18-T.	This work
pHL804	*gtt* sequences were excised from pHL801 with *Xba*I and *Spe*I and inserted into *Spe*I site of pMS82.	Guo et al., 2012
pHL805	*epi* sequences were excised from pHL802 with *Xba*I and *Spe*I and inserted into *Spe*I site of pHL804.	Guo et al., 2012
pHLJ811	*gdh-kre* sequences were excised from pHLJ810 with *Xba*I and *Spe*I and inserted into *Spe*I site of pHL805.	This work
pHLJ814	The PermE*-sfp sequence, flanked by *Xba*I and *Spe*I restriction sites, was synthesized and cloned into pMD18-T.	This work
pHLJ815	The PermE* -*sfp* sequence was excised from pHLJ814 with *Xba*I and *Spe*I and inserted into *Spe*I site of pHLJ811.	This work

### Construction of *Sac*. *spinosa* NRRL18395 Genomic BAC Library

The *Sac. spinosa* NRRL18395 genomic BAC library was constructed according to a standard protocol (Wang et al., 2005; Zhao et al., 2016). An overnight culture of *Sac. spinosa* NRRL18395 was harvested, embedded in agarose plugs, and the cells in the plugs were digested with lysozyme and proteinase K and then partially digested with *Sau*3AI. The ca. 150 kb genomic DNA fragments were fractionated by pulsed-field gel electrophoresis (PFGE), recovered, and ligated with *Bam*HI-linearized and dephosphorylated BAC vector pHL931. The ligation mixture was electroporated into *E. coli* DH10B competent cells and plated onto LB agar plates with apramycin (50 μg/mL) for an overnight incubation at 37°C. The resulting transformants were picked and stored in 96-well microtiter plates.

### Construction of Genomic Cosmid Library of *Sac. erythraea* HOE107

The genomic cosmid library of *Sac. erythraea* HOE107 was constructed as described previously (Gould et al., 1998). The genomic DNA of the bacterium was extracted and partially digested with *Mbo*I. The 35–45 kb fragments were isolated by PFGE, dephosphorylated, and ligated into the SuperCos 1 vector. The resulting ligation mixture was packaged into the λ phage, followed by phage transfection into the *E. coli* XL1-Blue MR strain. A cosmid, 6C9, was identified by PCR and end-sequencing as carrying a 40.9 kb *eryCI*-*eryCVI* region of the *ery* gene cluster.

### Establishment and Optimization of a Conjugal Transfer System

The mobilization of the BAC clones from the *E. coli* host to *Sac. erythraea* was accomplished using a triparental conjugation approach, including *E. coli* ET12567/pUB307 (helper), *E. coli* DH10B/BACs (donors), and *Sac. erythraea* SLQ185 (recipient). The *Sac. erythraea* spores (10^8^ cfu) were washed and resuspended in 250 μL 2 × YT broth (Kieser et al., 2000) at a concentration of 10^8^ per mL before incubation at 50°C for 10 min. *E. coli* DH10B containing conjugative BAC clones and *E. coli* ET12567/pUB307 were grown in LB separately to an OD_600_ of 0.6 at 37°C. These cells were washed twice with LB and resuspended together in 250 μL 2 × YT. Subsequently, the *E. coli* donor-helper mixtures were added to the spores, mixed thoroughly by pipetting, and spread on the solid conjugation media ESM; 2CM (Li and Piel, 2002); IWL4 (Min et al., 2003); or ISP4 (Choi et al., 2004). After an incubation of 16–20 h, each plate was flooded with trimethoprim (50 μg/mL) and apramycin (50 μg/mL) and incubated for 5 days at 34°C. Transfer frequency was then calculated as the ratio between the number of exconjugants on an antibiotic-selective plate and the number of recipient cells. The average frequency of three independent experiments was calculated.

### High-Throughput Transfer of the BAC Library Into *Sac. erythraea* SLQ185

The *Sac. erythraea* spores (recipient) and *E. coli* ET12567/pUB307 (helper) were prepared as described above and resuspended together in 500 μL 2 × YT. Then, the mixtures of recipient and helper cells were spread on ISP4 plates, followed by air-drying. The BAC library (conjugation donor) was inoculated into 96-well plates with antibiotic-selective LB (130 μl per well) at 37°C overnight, and then transferred to antibiotic-free LB and cultured for 4-6 h until the OD_600_ reached 0.4 to 0.6. The *E. coli* DH10B/BACs were then transferred with a 48-pin replicator onto the helper-recipient pre-coated plates and incubated at 34°C for 20 h. Then, the conjugation plates were flooded with apramycin and trimethoprim to a final concentration of 50 μg/ml and incubated at 34°C for another 5 days.

### Replacement of the *ery* Gene Cluster With the *attB^ϕ*C31*^*-*aadA-attB^ϕ*BT1*^* Cassette via Homologous Recombination

The *ery* gene cluster was replaced with an *attB^ϕ*C*31^*-*aadA*-*attB^ϕ*BT*1^* cassette via homologous recombination. Firstly, the *aadA* (aminoglycoside resistance gene) was amplified from pIJ4642 using primers aadA-F and aadA-R, which have, respectively, an *attB^ϕ*C*31^* and *attB^ϕ*BT*1^* sequence at the 5′-end. The resulting PCR product, *attB^ϕ*C*31^*-*aadA*-*attB^ϕ*BT*1^*, was cloned into pMD18-T vector (Takara) to generate pHLQ1. The *attB^ϕ*C*31^*-*aadA*-*attB^ϕ*BT*1^* cassette on pHLQ1 was amplified by PCR with primer pairs eryT-F/eryT-R, which harbor overhang regions complementary to the boundaries of the *eryAI*-*eryBIII* genomic region. Genes *eryAI*-*eryBIII* in cosmid 6C9 were then replaced with the *attB^ϕ*C*31^*-*aadA*-*attB^ϕ*BT*1^* cassette, using a λ-Red recombination protocol (Gust et al., 2003), to generate pHLQ2, which contains an *ery* gene cluster disruption cassette with an *attB^ϕ*C*31^*-*aadA*-*attB^ϕ*BT*1^* fragment flanked with a 5.1-kb upper homologous arm (UHA) and a 5.4-kb downstream homologous arm (DHA) homologous to the *ery* gene cluster. The *ery* disruption cassette was excised from pHLQ2 with *Xba*I and *Spe*I and inserted into the *Spe*I site of pOJ260 (Bierman et al., 1992) to generate the *ery* gene replacement construct pHLQ3. pHLQ3, which does not contain an autonomous replication region or integration locus that function in actinobacteria, was introduced into *Sac. erythraea* HOE107 by the triparental conjugation approach. Spectinomycin-resistant and apramycin-sensitive mutant strains were verified by PCR and sequence analysis, using primer pairs eryV1-F/eryV1-R, eryV2-F/eryV2-R, and eryV3-F/eryV3-R. The confirmed mutant strains were renamed *Sac. erythraea* SLQ185.

### Deletion of Genes *pkeA1–pkeA4* and the *rpp* Gene Cluster Mediated by the Combined CRISPR/Cas9-CodA(sm) Recombination System

For deletion of the *pke* gene cluster, we selected *pkeA1*-*pkeA4* between ORFs SACE_RS20070 and SACE_RS20095 (RefSeq NC_009142.1) as the knock-out target, using a CRISPR/Cas9-CodA(sm)-based approach (Zeng et al., 2015). A double-stranded DNA fragment encoding a small guide RNA (sgRNA) scaffold was obtained by annealing the 5′-phosphorylated oligonucleotides pkeG-F and pkeG-R. The DNA fragment was cloned into vector pWHU2653 (Zeng et al., 2015) at the *Bae*I restriction site to generate pHLJ67. Next, 2.1-kb UHA and 2.2-kb DHA sequences were amplified from *Sac. erythraea* HOE107 genomic DNA using the primer pairs pkeU-F/pkeU-R and pkeD-F/pkeD-R, respectively, and the two DNA fragments were joined together by overlapping PCR using the primer pair pkeU-F/pkeD-R. The PCR product was cloned into the pMD18-T TA cloning vector to generate pHLJ68, and then the UHA-DHA_pke_ region from pHLJ68 was cloned into pHLJ67 at the *Eco*RI-*Ase*I sites to generate the *pke* gene cluster replacement construct pHLJ69.

For deletion of the *rpp* gene cluster, we selected the *orfA*-*orfF* region (including *rppAB*) between SACE_RS06025 and SACE_RS06085 (RefSeq NC_009142.1) as the knock-out target, using the CRISPR-Cas9-based approach (Zeng et al., 2015). A double-stranded DNA encoding an sgRNA scaffold was obtained by annealing the 5′-phosphorylated oligonucleotides rppG-F and rppG-R. The DNA fragment was cloned into vector pWHU2653 via the *Bae*I restriction site to generate pHLJ61. The 2.2-kb UHA and 2.2-kb DHA sequences were amplified from *Sac. erythraea* HOE107 genomic DNA using the primer pairs rppU-F/rppU-R and rppD-F/rppD-R, respectively, and the two DNA fragments were joined together by overlapping PCR using the primer pair rppU-F/rppD-R. The PCR product was cloned into pMD18-T to generate pHLJ62, and then the UHA-DHA_rpp_ region from pHLJ62 was cloned into pHLJ61 at the *Eco*RI-*Ase*I sites to generate the *rpp* gene cluster replacement construct pHLJ63.

pHLJ69 and pHLJ63 were separately transformed into *Sac. erythraea* SLQ185 by *E. coli*-*Sac. erythraea* triparental conjugation. Independent apramycin-resistant exconjugants were streaked onto solid ESM medium, containing 800 μg/mL 5-fluorocytosine (5FC, for selection against the vector backbone) and 25 μg/mL nalidixic acid (for selection against *E. coli*), and then grown in the dark for 5 days. The 5FC-resistant colonies were replicated onto plates with or without apramycin to confirm plasmid loss. Genomic DNA of single apramycin-sensitive colonies was extracted and used to screen for gene replacement mutants by PCR. The *pkeA1–pkeA4* deletion mutant, named *Sac. erythraea* LJ161, was confirmed by PCR with primer pairs pkeV4-F/pkeV4-R and pkeV5-F/pkeV5-R and sequence analysis. The *rpp* deletion mutant, named *Sac. erythraea* LJ162, was confirmed by PCR with primer pairs rppV6-F/rppV6-R and rppV7-F/rppV7-R and sequence analysis.

### Construction of Integrative Plasmids Carrying Genes *gtt, epi, gdh-kre*, and *sfp*

The *gtt, epi*, and *gdh-kre* gene sequences were amplified from *Sac. spinosa* NRRL18395 genomic DNA using the primer pairs gtt-F1/gtt-R1, epi-F1/epi-R1, and GK-F1/GK-R1 respectively. The three PCR products were cloned into pMD18-T to generate pHL801, pHL802, and pHLJ810. The 1299-bp *Spe*I/*Xba*I fragment containing *gtt* from pHL801 was cloned into the *Spe*I site of pMS82 to give pHL804. The 1193-bp *Spe*I/*Xba*I fragment containing *epi* from pHL802 was cloned into the *Spe*I site of pHL804 to give pHL805. The 2459-bp *Spe*I/*Xba*I fragment containing *gdh-kre* from pHLJ810 was cloned into the *Spe*I site of pHL805 to give pHLJ811. The codon-optimized gene *sfp* was synthesized based on the protein sequence of Sfp of *Bacillus subtilis* (AEK64474.1), using the web server for codon optimization^1^. The strong, constitutive promoter *PermE*^∗^ (Bibb et al., 1985) was placed upstream of *sfp* to control *sfp* expression, and the *PermE*^∗^-*sfp* DNA fragment with flanking *Xba*I and *Spe*I sites was cloned into pMD18-T to generate pHLJ814. The 804-bp *Spe*I/*Xba*I restriction fragment containing *PermE*^∗^-*sfp* was excised from pHL814 and ligated with the *Spe*I-linearized pHLJ811 to give pHLJ815.

### Bioassay of *Sac. erythraea* HOE107 and SLQ185

Fresh spores of *Sac. erythraea* HOE107 and SLQ185 were spread onto EFM supplemented with 3 mM Fe^3+^. After culturing at 28°C for 7 days, agar plugs (8-mm diameter) were taken and put onto the surface of LB agar plates previously inoculated with an overnight culture (1:100) of either *Micrococcus luteus* or *B. subtilis*, followed by incubation overnight at 37°C.

### Fermentation Conditions, Extraction of Secondary Metabolites, and Measurement of Biomass Dry Weight

For erythromycin production, three pieces of culture lawn (ca. 1.5 cm^2^) were cut from the sporulating plates and inoculated into 25 mL of seed medium (0.5% glucose, 2.5% corn starch, 1% yeast extract, 1% whole-milk powder, 0.2% MgSO_4_⋅7H_2_O, pH 7.2) in a 250 mL flask and incubated at 28°C for 72 h on a rotary shaker at 250 rpm as described previously (Huang et al., 2016). Then, 2 mL of seed culture was inoculated in 30 mL of the fermentation medium EFM [4% cornstarch, 3% soybean flour, 3% dextrin, 0.2% (NH_4_)_2_SO_4_, 1% soybean oil, 6% CaCO_3_, pH7.2] (Li et al., 2013) in a 250 mL flask and incubated under the same conditions for 7 days. Erythromycin was extracted from the fermentation culture following the methods described previously (Le et al., 2001; Kirm et al., 2013). Briefly, the broth was adjusted to pH 10 and mixed with an equal volume of acetonitrile for 40 min. Then, 2 g NaCl was added per 10 mL broth, left to dissolve, and the acetonitrile phase was then separated by centrifugation.

For spinosad production, strains were cultured in seed medium as described above, and then 2 mL of cultured seed was inoculated into 30 mL of the fermentation media EFM or HJFM (9% glucose, 2% whole-milk powder, 2.5% cottonseed cake powder, 0.2% yeast powder, 0.1% lactic acid, 0.4% trisodium citrate, 0.2% K_2_HPO_4_, pH 7.2) (Huang et al., 2016) in a 250 mL flask under the same conditions for 10 days. After fermentation, 1 mL of each culture was extracted with 4 mL of methanol in an ultrasonic bath for 30 min, centrifuged, and the supernatant was analyzed by high-performance liquid chromatography (HPLC) to detect the spinosad production. At the meantime, each culture was sampled for measurement of the biomass dry weight: 1 mL culture was centrifuged at 9,000 rpm for 10 min to collect the pellet, which was hold in the desiccator at 75°C for 3 days to obtain a constant weight.

For actinorhodin production, strains were cultured in R3 agar medium (Shima et al., 1996) at 28°C for 7 days, and then 500 mg of each culture containing both bacteria and agar was taken from the plates and put into 1.5 mL Eppendorf tubes, followed by the addition of 500 μl of KOH or methanol to each tube. The tube contents were dispersed in a homogenizer with glass beads (0.1 mm in diameter) and centrifuged at 13,000 rpm for 10 min to remove the particulate matter and collect the liquid crude extract for the measurement of actinorhodin by optical absorbance.

### Chromatographic Analysis of Secondary Metabolite Production

The metabolite sample of erythromycin was applied to a Zorbax SB-C18 column (5 μm particle size, 4.6 × 250 mm, Agilent, Germany) installed on the Agilent 1260 Infinity II LC system. Isocratic elution was applied with the mobile phase consisting of 40% 50 mM K_2_HPO_4_ (pH adjusted to 9 with diluted phosphoric acid) and 60% acetonitrile. After injection of 10 μl of the sample solution, the HPLC system was operated at a flow rate of 0.6 mL/min, with a total run time of 50 min. The column temperature was set at 60°C and the detection wavelength at 206 nm as described previously (Kirm et al., 2013). Reference substance erythromycin A (purity > 98%) was obtained from Aladdin Bio-Chem Technology Co. (Shanghai, China). A standard solution was prepared by dissolving 5 mg of erythromycin in 5 mL of ethanol.

The metabolite sample of spinosad was analyzed by liquid chromatography/mass spectrometry (LC/MS) using the Agilent 1260 Infinity II LC system coupled to Agilent 6470 triple quadrupole mass spectrometry (MS) instruments. The separation was performed on a Zorbax SB-C18 column (5 μm particle size, 4.6 × 250 mm, Agilent) and elution was performed with an isocratic mobile phase consisting of methyl-acetonitrile-0.05% sodium acetate with the volume ratio of 45:45:10, at a flow rate of 0.35 mL/min, and detected at 250 nm as described previously (Huang et al., 2016). The MS analysis was conducted in the positive ion mode with capillary voltage and nozzle voltage set at 3,500 and 500 V, respectively. The gas temperature was set to 300°C at a flow rate of 5 L/min. Sheath gas temperature was set to 250°C at a flow rate of 11 L/min. Spinosad was monitored using multiple reaction monitoring transitions at 732.5 to 142.1 and 98.1 *m/z* for spinosyn A, and 746.5 to 142.1 and 98.1 *m/z* for spinosyn D with positive electrospray ionization. The obtained data were evaluated by Agilent MassHunter workstation software (Agilent Technologies). Data are representative of three independent experiments. Reference substance spinosad (66% spinosyn A and 28% spinosyn D) was obtained from Dr. Ehrenstorfer GmbH (Augsburg, Germany). A standard solution was prepared by dissolving 5 mg of spinosad in 5 mL of methanol.

For the metabolite sample of actinorhodin, the absorbance at 640 nm was read using a microplate reader (Bioteke Corporation), and the background absorbance of the culture extraction in the absence of bacteria was subtracted. Data are representative of three independent experiments.

## Results

### Replacement of the Erythromycin Biosynthetic Gene Cluster in *Sac. erythraea* HOE107 via a Conventional Homologous Recombination Approach

**FIGURE 1:**
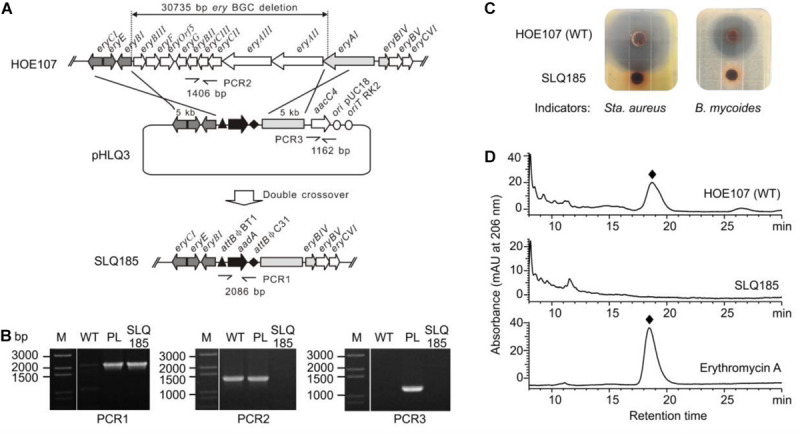
Replacement of the erythromycin BGC on the *Sac. erythraea* chromosome with the *attB^ϕ*C*31^-aadA-attB^ϕ*BT*1^* cassette. **(A)** Schematic representation of the homologous recombination (double crossover) between the wild-type *ery* locus and the gene replacement vector pHLQ3 to give the gene replacement mutant SLQ185. PCR1, PCR2, and PCR3 are PCR reactions designed for the verification of the mutant. **(B)** Verification of the genotype of the mutant SLQ185 by PCR. M, molecular marker; WT, *Sac. erythraea* HOE107; PL, plasmid pHLQ3; SLQ185, *Sac. erythraea* SLQ185. **(C)** Analysis of the antibacterial activity of the *Sac. erythraea* strains by growth inhibition of *Sta. aureus* (left) and *B. mycoides* (right). **(D)** HPLC analysis of the production of erythromycin A in *Sac. erythraea* HOE107 and SLQ185. The bottom profile shows the erythromycin A standard.

*Saccharopolyspora erythraea* HOE107 has been used for the industrial production of erythromycin. It is an erythromycin-overproducing descendent of *Sac. erythraea* NRRL 23338 obtained by mutagenesis and selection. Genome sequencing of *Sac. erythraea* HOE107 revealed multiple point mutations, including a nonsense mutation in a polyketide biosynthetic gene cluster *pks3* as reported in *Sac. erythraea* Px (Peano et al., 2012; Li et al., 2013). To re-engineer this strain for the heterologous production of other polyketides, we replaced a 30.7-kb fragment of the erythromycin BGC in *Sac. erythraea* HOE107 by an *attB^ϕ*C*31^*-*aadA*-*attB^ϕ*BT*1^* cassette via double crossover between a gene replacement construct pHLQ3 and the *Sac. erythraea* HOE107 chromosome (Figure 1A). Intergenus conjugation between *E. coli* DH10B/pHLQ3 and *Sac. erythraea* HOE107 and subsequent screening of the resulting exconjugants gave rise to 20 spectinomycin-resistant and apramycin-sensitive colonies. The genotype of SLQ185 was verified by PCR, using primer pairs eryV1-F/R to confirm that the spectinomycin resistance gene *aadA* was successfully inserted at gene cluster *ery* (Figures 1A,B); using primer pairs eryV2-F/R to confirm deletion of the partial gene cluster *ery*; and using primer pairs eryV3-F/R to confirm the loss of the apramycin resistance gene (*aacC4*), indicating that the plasmid pHLQ3 was eliminated (Figure 1B). However, PCR indicated that only three of the 20 Spc^*R*^Apr^*S*^ colonies were gene replacement mutants, in which nine erythromycin biosynthetic genes, i.e., *eryAII*-*eryBIII*, and part of *eryAI* were deleted. One of these mutants was named SLQ185.

Since erythromycin produced by *Sac. erythraea* displays broad-spectrum activity against Gram-positive organisms at very low concentrations, we tested the antibacterial phenotype of the gene replacement mutant strain *Sac. erythraea* SLQ185. The wild-type strain and SLQ185 were cultivated on EFM agar medium, which was supplemented with 3 mM Fe^3+^ to suppress the possible production of erythrochelin, a 2,5-diketopiperazine siderophore that shows weak antibacterial activity against Gram-positive organisms (Lazos et al., 2010). As shown in Figure 1C, the plugs of cultivated *Sac. erythraea* HOE107 produced large zones of inhibition against *Sta. aureus* or *B. subtilis* whereas the plugs of SLQ185 did not produce any inhibition zone, suggesting the loss of erythromycin production. HPLC analysis confirmed that the erythromycin biosynthesis was completely abolished in the gene replacement mutant SLQ185 (Figure 1D).

### Deletion of the *pke* or *rpp* Gene Cluster From *Sac. erythraea* SLQ185 Using an Established CRISPR/Cas9-CodA(sm) Combined Homologous Recombination System

As one study indicated that the type I PKS BGC *pke* and the type III PKS BGC *rpp* were actively expressed in *Sac. erythraea* and competed with erythromycin production (Li et al., 2013), we decided to delete these two PKS BGCs from the SLQ185 genome to make a clean metabolic background and to avoid potential substrate competition between endogenous and heterologous biosynthetic pathways. To construct the deletion mutants in an efficient way, we chose the CRISPR/Cas9-CodA(sm) gene knockout system, which worked well in *Streptomyces* spp. (Zeng et al., 2015). The CRISPR/Cas9-based RNA-guided DNA digestion was utilized to stimulate homologous recombination between the target chromosome locus and the homologous repair template pairs provided by a gene-targeting construct. The counterselection marker *codA*(sm) on the plasmid, which confers 5-fluorocytosine (5FC) sensitivity to the host cell, was used for the selection of a recombinant that had lost the target genes and the plasmid backbone (Zeng et al., 2015).

**FIGURE 2:**
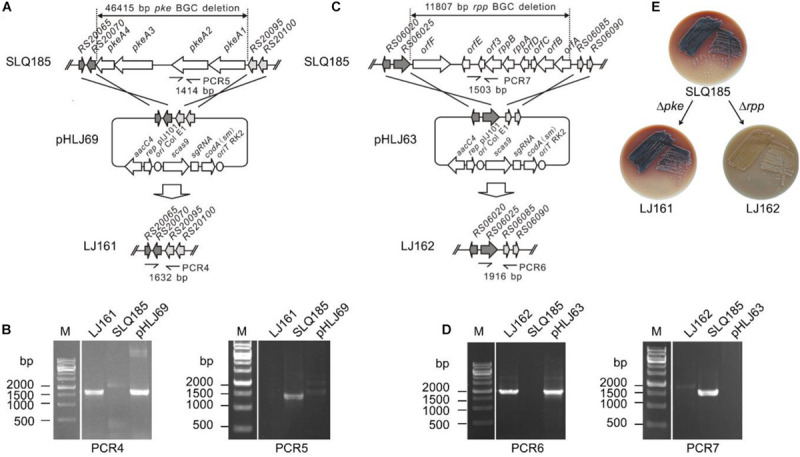
CRISPR/Cas9-CodA(sm)-mediated deletion of the *pke* and *rpp* PKS biosynthetic gene clusters. **(A)** Schematic representation of the homologous recombination between the *pke* locus and the gene deletion construct pHLJ69 generating the gene deletion mutant LJ161. PCR4 and PCR5 are PCR reactions designed for the verification of the mutant. **(B)** Verification of the genotype of LJ161 by PCR. M, molecular marker. **(C)** Schematic representation of the homologous recombination between the *rpp* locus and the gene deletion construct pHLJ63 generating the gene deletion mutant LJ162. PCR6 and PCR7 are PCR reactions designed for the verification of the mutant. **(D)** Verification of the genotype of LJ162 by PCR. **(E)** Photographs showing the surface growth and pigmentation production of the parental strain and deletion mutants. Photos were taken after 7 days of cultivation on ESM medium at 34°C.

To delete the PKS genes *pkeA1–pkeA4* from the *pke* BGC, the pHLJ69 was constructed based on the CRISPR/Cas9-CodA vector pWHU2653 and introduced into SLQ185. pHLJ69 contains the codon-optimized CRISPR-Cas9 gene *scas9*; a sgRNA gene targeting the *pke* BGC; the *codA*(sm) gene; and two DNA fragments of 2.1 and 2.2 kb, which were homologous to the flanking regions of the *pke* BGC and were used as repair templates (Figure 2A). After conjugal transfer of pHLJ69 into SLQ185 and subsequent screening, 20 resulting 5FC-resistant and apramycin-sensitive colonies were randomly picked and tested by PCR. The PCR results confirmed that all colonies were *pkeA1-pkeA4* deletion mutants, one of which is named *Sac. erythraea* LJ161 and shown in Figure 2B.

To delete the *rpp* BGC, the *rpp*-targeting plasmid pHLJ63, containing two 2.2-kb homologous repair arms, was constructed and introduced into *Sac. erythraea* SLQ185 by intergenus conjugation (Figure 2C). Twenty 5FC-resistant and apramycin-sensitive colonies were randomly selected and all were confirmed by PCR to be *rpp* deletion mutants, one of which is named *Sac. erythraea* LJ162 and shown in Figure 2D. In contrast to the *pkeA1*-*pkeA4*-deletion strain *Sac. erythraea* LJ161, which showed no obvious difference in growth or colony morphology compared with the parental strain *Sac. erythraea* SLQ185, the *rpp* mutant strain exhibited an albino phenotype and failed to produce the diffusible brown pigment characteristic of *Sac. Erythraea* when grown on solid ESM medium (Figure 2E).

### Construction of a BAC Library With Large Size Inserts and Conjugal Transfer of BAC Clones and Library Into *Sac. erythraea* SLQ185

**FIGURE 3:**
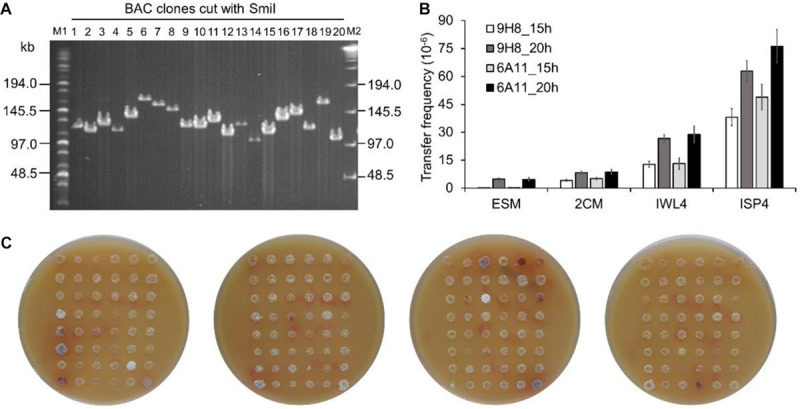
Transfer of large size BAC clones into *Sac. erythraea*. **(A)** Analysis of the insert size of *Sac. spinosa* NRRL18395 BAC clones by PFGE. M1, middle range PFG marker (NEB); M2, lambda ladder PFG marker (NEB); lanes 1–20, recombinant DNA digested with *Smi*I and obtained from alkaline lysis minipreps of randomly picked colonies. **(B)** Conjugation transfer of BAC clones 9H8 and 6A11 into *Sac. erythraea* SLQ185 evaluated on four types of agar media. The conjugation plates were incubated for 15 or 20 h before overlaying with antibiotics. Data are from three biological replicates. **(C)** High-throughput conjugation transfer of the BAC library into *Sac. erythraea* SLQ185 on the ISP4 agar plates. Photos of the conjugation plates were taken after 5 days of prolonged cultivation at 34°C after flooded with antibiotics for the selection of exconjugants. Spots indicate the exconjugants of corresponding clones of the *Sac. spinosa* genomic BAC library.

To test whether BAC clones harboring large size inserts could be efficiently transferred into the engineered *Sac. erythraea* strains from *E. coli*, we first constructed a BAC library using large fragments of genomic DNA from *Sac. spinosa* NRRL18395, the native producer of the macrolide polyketide insecticide spinosad. An integrative BAC vector pHL931 (Zhao et al., 2016), which contains an *attP-int* locus of phage ϕC31, was used as the cloning vector. All BAC clones would harbor the *attP^ϕ*C*31^-int* locus for mediating their own integration into actinomycete chromosomes at the *attB^ϕ*C*31^* site via site-specific recombination. The resultant BAC library had 960 clones and an average insert size of 110-kb (Figure 3A).

Two BAC clones, *E. coli* DH10B/9H8 and DH10B/6A11, which have sizes of 140 kb and 124 kb respectively, were randomly selected as the donors to explore a triparental conjugation protocol with *E. coli* ET12567/pUB307 as the helper and *Sac. erythraea* SLQ185 as the recipient. The donor, helper, and recipient cells were mixed and spread on four types of agar media (2CM, ISP4, IWL4 and ESM) separately and incubated for 15 or 20 h before overlaying with antibiotics for the selection of exconjugants. Two crucial parameters of conjugal transfer, i.e., the conjugation media and the time point for antibiotic overlay, were evaluated in these experiments. Of the media tested, ISP4 agar produced about ten times more exconjugants than 2CM and ESM did. A longer pre-incubation time (20 h) before antibiotic overlaying also led to slightly more exconjugants (*p* = 0.0037). The optimal conditions resulted in a high frequency of conjugal transfer of BAC clones from *E. coli* to *Sac. erythraea* SLQ185 (6.3 × 10^–5^ and 7.6 × 10^–5^ exconjugants/recipient) (Figure 3B). Under the same conditions, the smaller integrative vectors pSET152 (5.7 kb) and pHL931 (16.7 kb) gave two orders of magnitude more exconjugants than did these two BAC clones. When the SLQ185-derived strain LJ161 was used as the recipient, similar conjugation frequencies were observed. Since no spores were formed on the aerial hyphae of *rpp*-deletion strain *Sac. erythraea* LJ162 (Figure 2E), we had to use mycelium without heat-shock treatment instead of heat-shocked spores as the recipient during conjugation transfer, which reduced the frequency of conjugation slightly. We then tested the engineered strain *Sac. erythraea* SLQ185 as the recipient in high-throughput triparental conjugation with the BAC library. As shown in Figure 3C, the arrayed BAC genomic library of *Sac. spinosa* NRRL18395 was effectively transferred into SLQ185, in that all BAC clones gave growth spots consisting of exconjugants.

### Heterologous Expression of the Spinosad BGC From *Sac. spinosa* in the Engineered *Sac. erythraea* Strains

Spinosad is a mixture of spinosyns A and D produced by a type I PKS gene cluster (*spn*) from *Sac. spinosa* NRRL 18395. Most of the genes involved in spinosad biosynthesis are located in this cluster, which spans a 80-kb region, except for the four rhamnose biosynthetic genes *gtt*, *epi*, *gdh*, and *kre*, which are dispersed in the genome (Waldron et al., 2001). The *sfp* gene, which codes for a 4′-phosphopantetheinyl transferase, also contributes to spinosad synthesis, as introduction of this gene increased the heterologous production of spinosad in *Sac. erythraea* (Huang et al., 2016).

To express the spinosad biosynthetic pathway in our engineered *Sac. erythraea* strains, we screened the BAC library and isolated 3H2, a BAC clone harboring a 128-kb genomic insert covering the entire 80-kb spinosad BGC. We also constructed an *attP^ϕ*BT*1^*-based integrative plasmid, pHLJ815, carrying four rhamnose biosynthetic genes and a synthetic *sfp* gene. The *attP^ϕ*C*31^*-based BAC clone 3H2 and the *attP^ϕ*BT*1^*-based plasmid pHLJ815 were transferred into *Sac. erythraea* strains SLQ185, LJ161, and LJ162 for the heterologous expression of spinosad.

**FIGURE 4:**
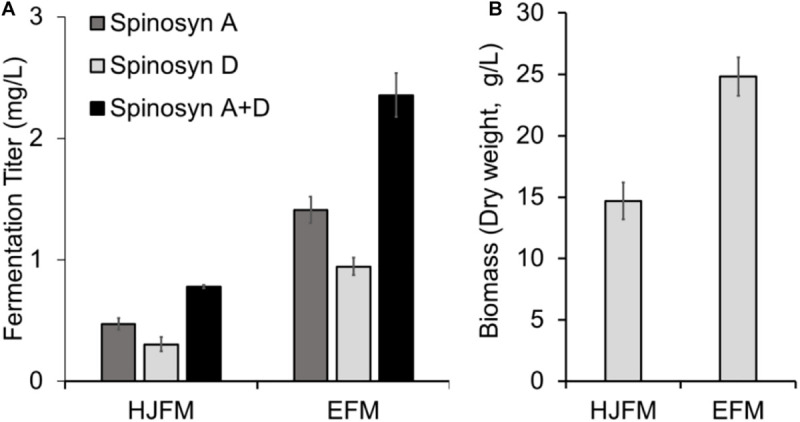
Heterologous production of spinosad by *Sac. erythraea* SLQ185/3H2/pHLJ815 in two types of fermentation media. **(A)** Production of spinosad in HJFM and EFM media. **(B)** Bacterial biomass. The dry weight of SLQ185**/**3H2**/**pHLJ815 was measured following growth in the two types of media. Data are from three biological replicates.

Firstly, we tested the effect of fermentation media on the heterologous production of spinosad. SLQ185/3H2/pHLJ815 was fermented in the erythromycin industrial fermentation medium (EFM) or HJFM, a medium previously used for the heterologous expression of spinosad (Huang et al., 2016). LC/MS analysis of the extracts of the fermented cultures showed that spinosad was produced in both media, although the yield of spinosad (i.e., sum of spinosyns A and D) with EFM was 102% higher than with HJFM (*p* = 0.00011) (Figure 4A). The increase of spinosad yield was partially due to the increase in dry weight, with EFM producing 69% (*p* = 0.0012) more biomass when compared with HJFM (Figure 4B).

**FIGURE 5:**
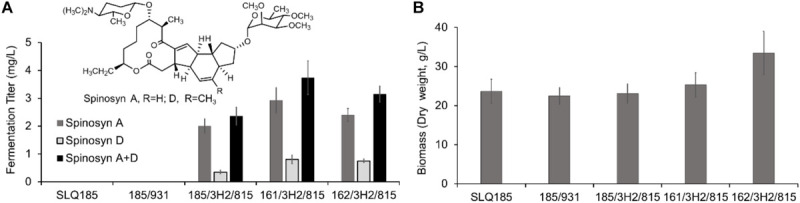
Heterologous production of spinosad by *Sac. erythraea* strains SLQ185, LJ161, and LJ162 carrying 3H2 and pHLJ815. 3H2 contains the 80-kb *spn* BGC, and pHLJ815 contains the rhamnose biosynthetic genes (*gtt, epi, gdh, kre*) and *sfp*. **(A)** Fermentation titer of spinosyns A and D. **(B)** Bacterial biomass. The dry weight of *Sac. erythraea* strains was measured following growth in the fermentation medium EFM for 10 days. Data are from three biological replicates.

To evaluate the impact of the *pke* or *rpp* deletion on the production of spinosad, BAC clone 3H2 and pHLJ815 were transferred into the engineered strains *Sac. erythraea* strains LJ161 and LJ162, generating LJ161/3H2/pHLJ815 and LJ162/3H2/pHLJ815, respectively. We found that the yield of spinosad in LJ161/3H2/pHLJ815 and LJ162/3H2/pHLJ815 improved slightly, by 58% (*p* = 0.0070) and 34% (*p* = 0.011), respectively, when compared to SLQ185/3H2/pHLJ815. The highest yield of spinosad was produced by LJ161/3H2/pHLJ815, reaching a level of 3.73 mg/L (Figure 5A). In addition, the dry weight of LJ162/3H2/pHLJ815 was improved by 45% (*p* = 0.0078) in comparison with that of SLQ185/3H2/pHLJ815 (Figure 5B).

### Heterologous Expression of the Actinorhodin BGC From *S. coelicolor* in the Engineered *Sac. erythraea* Strains

**FIGURE 6:**
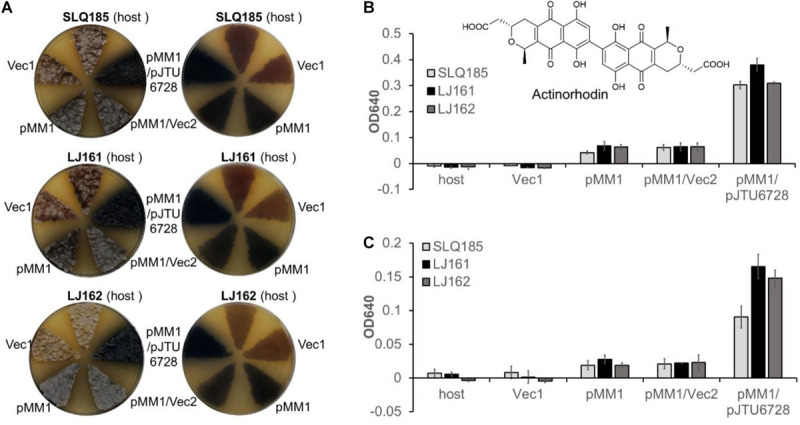
Heterologous production of actinorhodin by *Sac. erythraea* strains SLQ185, LJ161, and LJ162 carrying pMM1and pJTU6728. **(A)** Heterologous production of the blue pigment actinorhodin on R3 agar plates. The surfaces (left) and the backs (right) of the culture plates are shown. Vec1, the vector pMM1; Vec2, the vector pMS82. **(B,C)** Quantification of actinorhodin production in R3 medium. The cultures were extracted by either methanol **(B)** or 1 M KOH **(C)**. The optical density (OD) was determined at 640 nm. Data are from three biological replicates.

Actinorhodin is synthesized from acetyl-CoA and malonyl-CoA by a type II PKS encoded by the *act* BGC in *S. coelicolor* A3(2) (Bystrykh et al., 1996). To assess the effects of the deletion of PKS BGCs (*pkeA1-pkeA4* and *rpp*) on the heterologous biosynthesis of actinorhodin, the previously described *attP^ϕ*C*31^*-based integrative plasmid pMM1 (Zhou et al., 2012), which contains the entire actinorhodin BGC, was introduced into *Sac. erythraea* SLQ185, LJ161, and LJ162 to generate SLQ185/pMM1, LJ161/pMM1, and LJ162/pMM1, respectively. When the exconjugants were cultivated on solid R3 medium, the heterologous expression of the actinorhodin BGC resulted in the observable production of the blue-pigmented actinorhodin (Figure 6A). To compare actinorhodin production quantitatively, the cultures were extracted with either 1 M KOH as described previously (Bystrykh et al., 1996) or methanol, and the extracts were monitored by UV absorbance at 640 nm. We found that, from the same culture, extraction with methanol produced 1–2 times more blue pigment than did alkaline extraction (*p* < 0.001) (Figures 6B,C), suggesting that the methanol extraction data were more representative of the actual yields in the cells. Additionally, in the methanol extraction data, the yield of blue pigment from LJ161/pMM1 and LJ162/pMM1 was improved slightly (52%, *p* = 0.059; and 43%, *p* = 0.025, respectively) in comparison with that of SLQ185/pMM1 (Figure 6B).

We have previously demonstrated that pJTU6728, an *attP^ϕ*BT*1^*-based integrative plasmid carrying the transcription factor gene *nusG*, the global regulator gene *afsS*_*cla*_, and the two drug-efflux pump genes *mdfA*_*co*_ and *lrmA*_*co*_, increased the heterologous production of secondary metabolites in an *S. lividans* host (Peng et al., 2018). To assess the effects of pJTU6728 on the heterologous production of actinorhodin in *Sac. erythraea*, pJTU6728 and the empty vector pMS82 were individually introduced into SLQ185/pMM1, LJ161/pMM1, and LJ162/pMM1 and integrated into the host chromosome via the phage ϕBT1 *att*/*int* system. The yield of actinorhodin in the pJTU6728-containing strains SLQ185/pMM1/pJTU6728, LJ161/pMM1/pJTU6728, and LJ162/pMM1/pJTU6728 was 4.9, 5.9, and 4.8 times higher than in the corresponding vector control strains SLQ185/pMM1/pMS82, LJ161/pMM1/pMS82, and LJ162/pMM1/pMS82 (*p* < 0.0001) (Figure 6B). In addition, compared with the yield in SLQ185/pMM1/pJTU6728, the yield of blue pigment in the *pke* deletion mutant LJ161/pMM1/pJTU6728 was improved by 25% (*p* = 0.0089) in the methanol extraction and 82% (*p* = 0.0060) in the KOH extraction (Figures 6B,C).

## Discussion

Heterologous-expression hosts derived from rare actinomycetes are valuable for genome mining of bioactive natural products. Here, we described the optimization and application of the erythromycin-overproducing bacterium *Sac. erythraea* HOE107 as a host for the heterologous expression of polyketide BGCs. In our study, we disrupted the erythromycin biosynthetic PKS gene cluster and replaced it with two phage integration (*attB*) sites from the actinomycete phages ϕC31 and ϕBT1, yielding the gene replacement strain *Sac. erythraea* SLQ185, which harbors an *attB^ϕ*C*31^-aadA-attB^ϕ*BT*1^* in place of the *ery* BGC. Two different *attB* sites were considered a useful modification since one site (*attB^ϕ*C*31^*) served as the integration site for the heterologous BGC, and the other (*attB^ϕ*BT*1^*) provided an integration site for beneficial supplemental factors, such as pJTU6728 and pHLJ815, to increase the production of actinorhodin and spinosad, respectively. We also applied a reported CRISPR/Cas9-CodA(sm) combined recombination system to delete the *pke* BGC, encoding a type I PKS, or the *rpp* BGC, encoding a type III PKS, from *Sac. erythraea* SLQ185, producing strains *Sac. erythraea* LJ161 and LJ162, respectively. Our results demonstrated that the CRISPR/Cas9-CodA(sm) combined homologous recombination system substantially improves the efficiency of gene replacement in *Sac. erythraea*.

Nine erythromycin biosynthetic genes, including *eryAII*, *eryAIII* (with a type I thioesterase domain), and *eryORF5* (coding for a type II thioesterase, Hu et al., 2003), were deleted from the erythromycin overproducing strain. The 1.4-kb 3′-terminal part of *eryAI* was also deleted. The remaining parts of EryAI protein expressed in the strains would not load and release polyketide building blocks due to the lack of both type I and II thioesterases. Therefore, the deletion of *ery* BGC in this study would save biosynthetic substates for the heterologous expression of polyketide BGCs. Although the *pke* BGC was actively expressed in an erythromycin-producing strain of *Sac. erythraea* (Li et al., 2013), extensive searches using 50 different types of solid and liquid media have not detected the products of the *pke* BGC (Boakes et al., 2004; Oliynyk et al., 2007). In this study, the deletion of the *pke* BGC slightly increased the production of both spinosad and actinorhodin, indicating that the multifunctional PKS machinery encoded by the *pke* BGC was functional in these conditions and that it competed with the introduced heterologous PKS pathways for precursors such as malonyl-CoA. The *rpp* BGC is another actively expressed PKS gene cluster in *Sac. erythraea* (Cortés et al., 2002). Deletion of the *rpp* BGC abolished the production of the brown pigment associated with this strain, which also alleviated substrate competition for polyketide production.

We also established a highly efficient conjugation protocol for transferring large-sized BAC clones into *Sac. erythraea* strains. Based on the optimized method, the arrayed BAC library was effectively transferred into *Sac. erythraea* SLQ185 using the massive triparental conjugation approach. We successfully expressed the spinosad BGC from *Sac. spinosa* and the actinorhodin BGC from *Streptomyces*, as indicated by the substantial production of the blue pigment in strains containing pJTU6728; these findings suggest that the engineered *Sac. erythraea* strains can serve as heterologous hosts in function-driven, genome-mining approaches, e.g., LEXAS (Xu et al., 2016), for the discovery of cryptic and new antibiotics from *Streptomyces* and rare actinomycetes.

In summary, we modified the erythromycin-producing strain *Sac. erythraea* into a heterologous host with a cleaner, less competitive metabolic background and an amendable genetic manipulation system, and demonstrated its utilization for the heterologous expression of polyketide BGCs from *Streptomyces* and *Sacchropolyspora*.

## Data Availability Statement

The original contributions presented in the study are included in the article/supplementary material, further inquiries can be directed to the corresponding author.

## Author Contributions

MT, JL, and ZD were responsible for the original concept and designed the experiments. MT, JL, XP, and YW analyzed the data. JL, QL, ZZ, LC, WH, JH, and KL performed the experimental work. JL and MT wrote the manuscript. All the authors read and approved the final manuscript.

## Conflict of Interest

The authors declare that the research was conducted in the absence of any commercial or financial relationships that could be construed as a potential conflict of interest.

## Footnotes

1 http://www.jcat.de
